# Supplementary material for: Newly Obtained Apple Pectin as an Adjunct to Irinotecan Therapy of Colorectal Cancer Reducing E. coli Adherence and β-Glucuronidase Activity
Source: Cancers (Basel). 2021 Jun 12;13(12):2952. doi: 10.3390/cancers13122952 (PMC8231545; doi:10.3390/cancers13122952)
Supplement: Supplementary file 1 [file cancers-13-02952-s001.zip › cancers-1224829-supplementary.pdf]

cancers

Supplementary Materials

Newly-obtained apple pectin as an adjunct to irinotecan therapy of colorectal cancer reducing *E. coli* adherence and β-glucuronidase activity

Anna Palko-Łabuz, Jerzy Maksymowicz, Beata Sobieszczańska, Agnieszka Wikiera, Magdalena Skonieczna, Olga Wesołowska and Kamila Środa-Pomianek

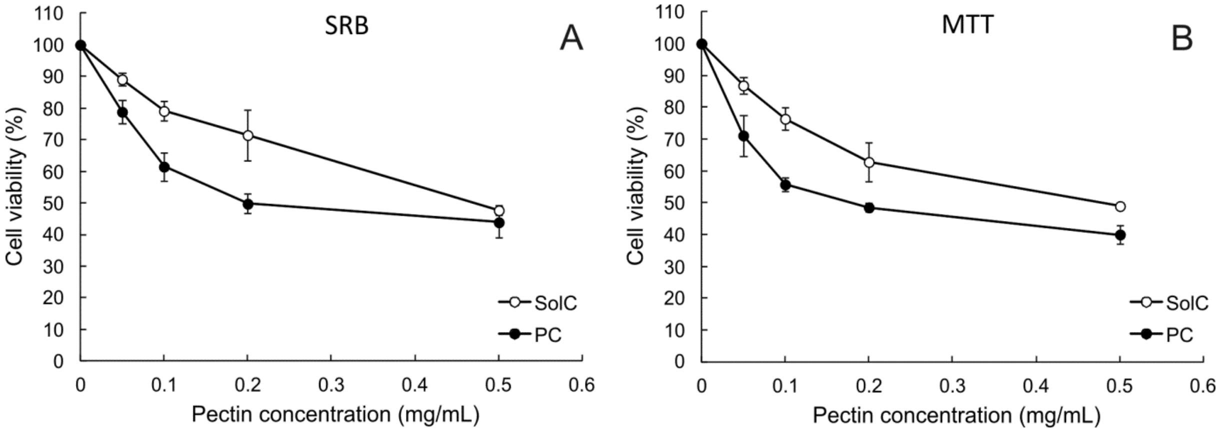

Figure S1. SRB (**A**) and MTT (**B**) cytotoxicity assay of pectins in Caco-2 cells (incubation time 48 hours). The means of three experiments ± SD are presented (* $p < 0.05$). Statistical significance was checked between the studied probes and controls (no pectin). In panels A and B all measurements were significantly different from the control (not marked on the graph for the sake of clarity).

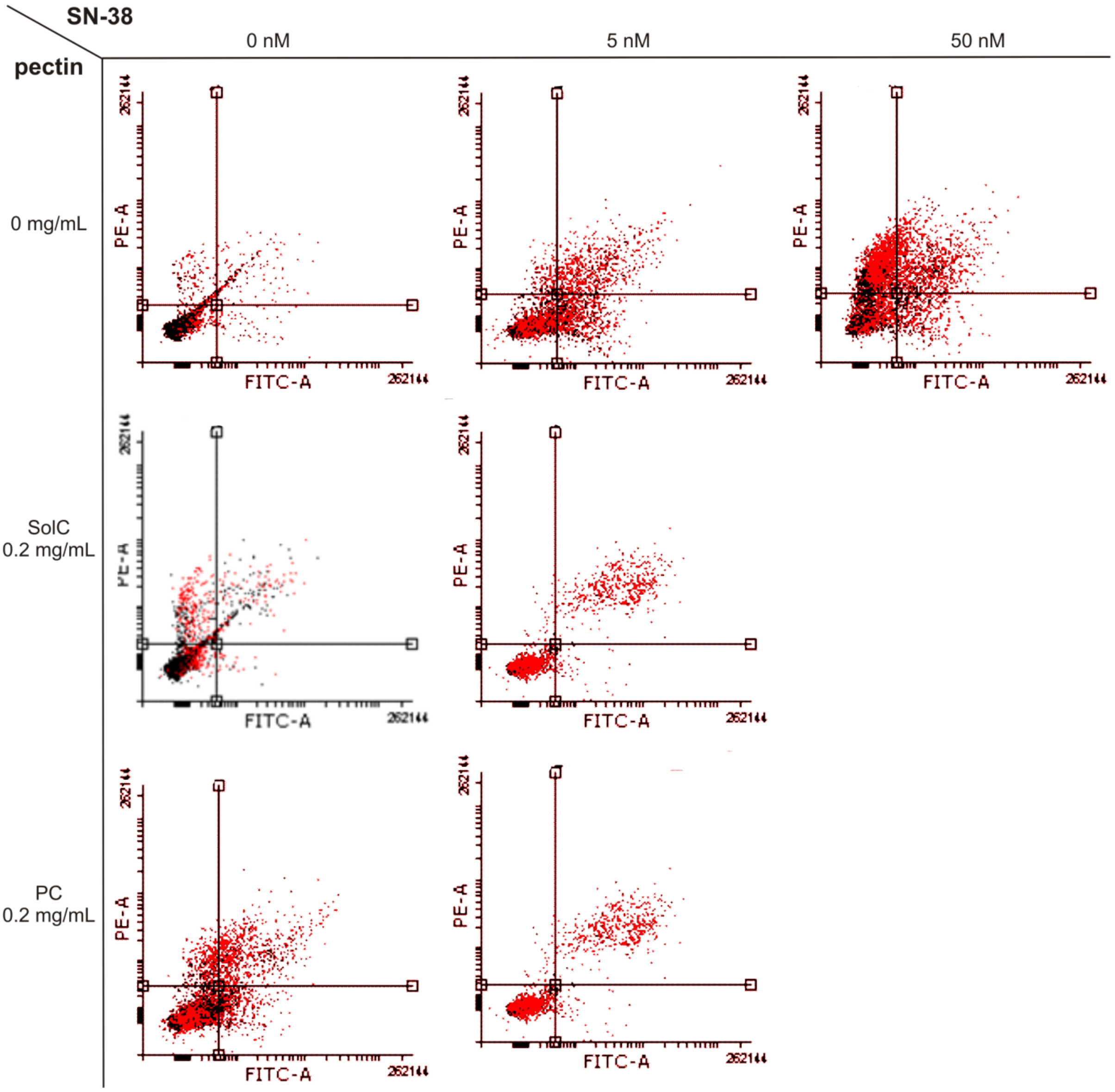

Figure S2. Typical dot plots of Annexin V/PI double staining apoptosis assay in HCT 116 cells. Cells were recognized as viable (Annexin-V and PI negative), apoptotic (Annexin-V positive and PI negative), and necrotic (Annexin-V and PI positive) based on the measurement of cell-associated fluorescence of FITC-Annexin-V conjugate and PI.

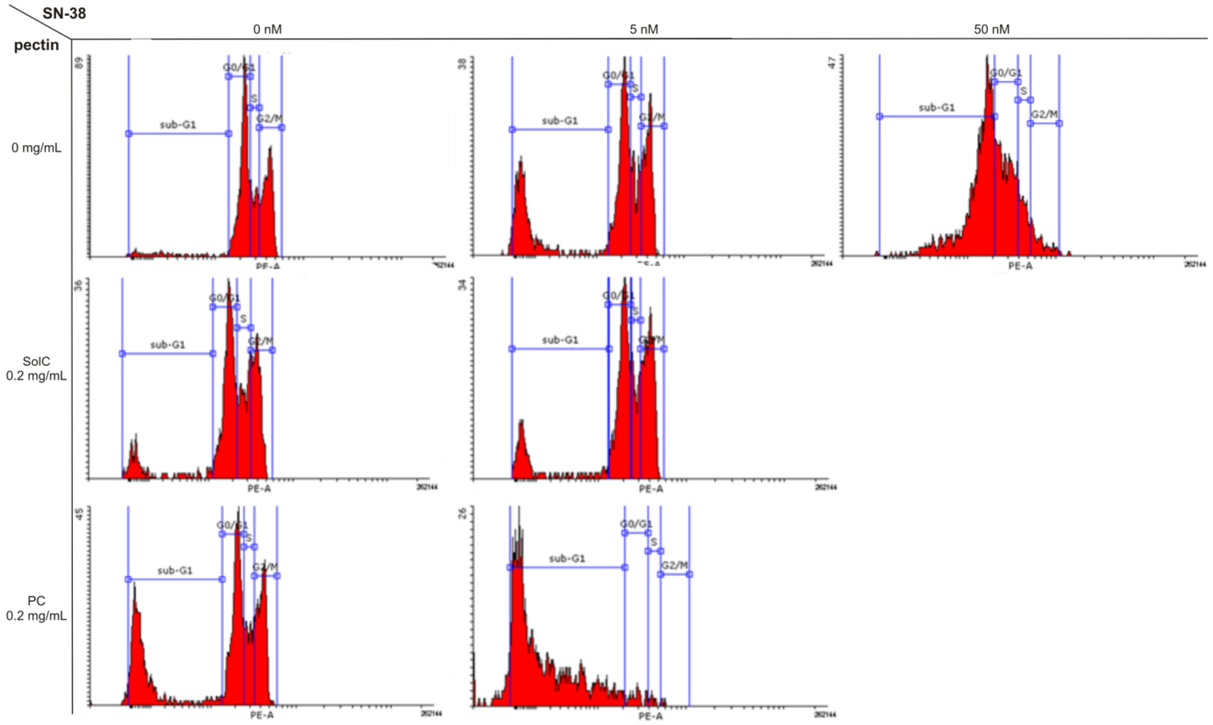

Figure S3. Typical histograms of DNA content (stained with PI) in HCT116 cells treated with 0.2 mg/mL of pectins and/or SN-38 for 48 hours. Sub-G_1 population—dead cells, G_0/G_1—mononuclear cells, S—DNA replication, G_2/M—mitosis.

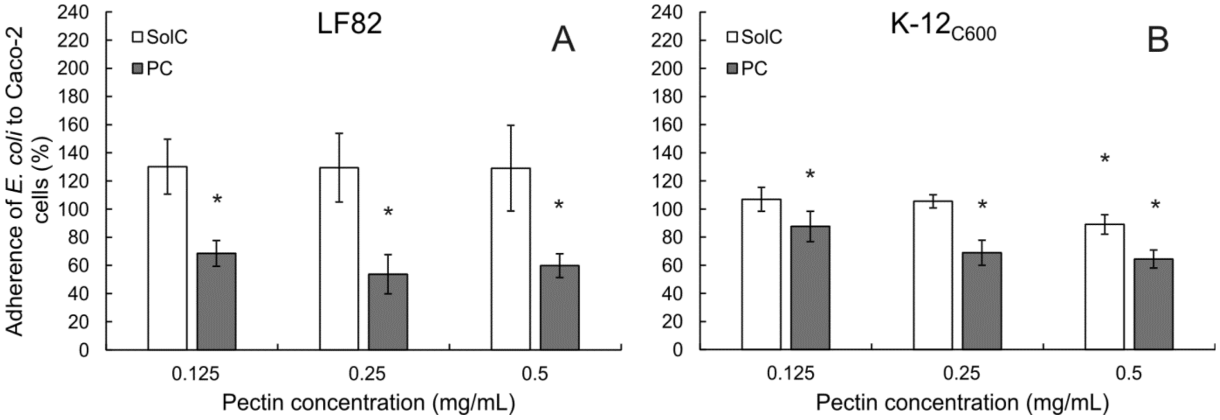

Figure S4. *E. coli* LH82 (**A**) and K-12_{C600} strains (**B**) adherence to Caco-2 cells in the presence of pectins during 2 hours of incubation. The means of three experiments ± SD are presented (* $p < 0.05$). Statistical significance was checked between the studied probes and controls (no pectin) assumed to be 100%.

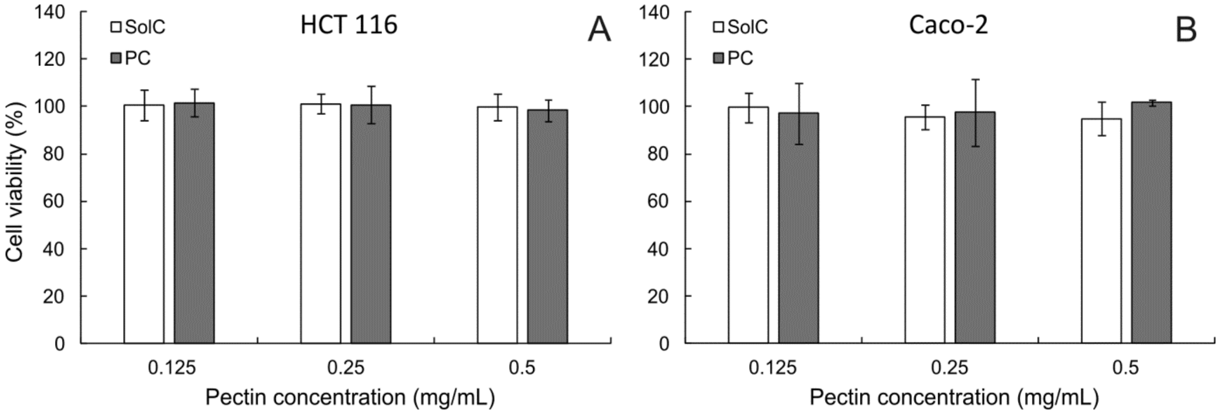

Figure S5. MTT (**B**) cytotoxicity assay of pectins in HCT 116 (**A**) and Caco-2 cells (**B**) during 2 hours of incubation. The means of three experiments ± SD are presented (* $p < 0.05$). Statistical significance was checked between the studied probes and controls (no pectin) assumed to be 100%.

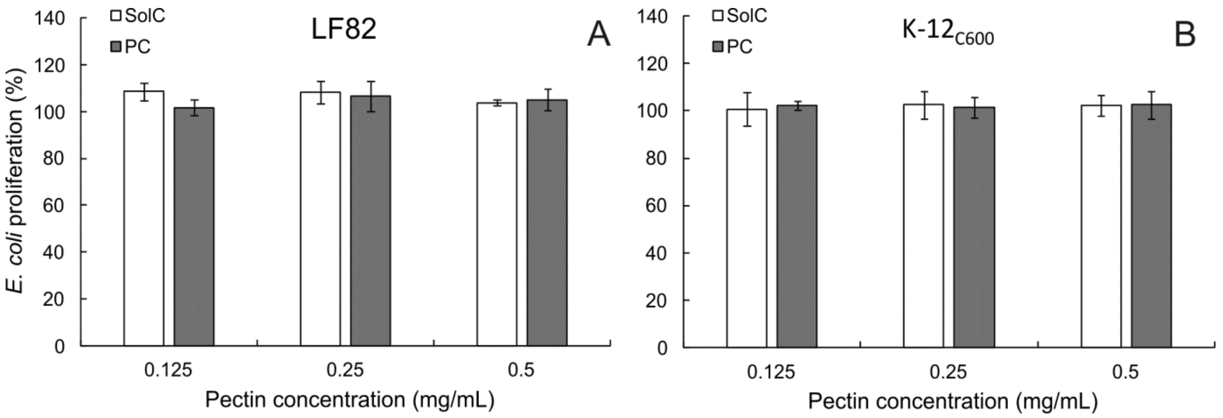

Figure S6. *E. coli* LH82 (**A**) and K-12$_{C600}$ strains (**B**) proliferation during 2 hours of incubation in the presence of pectins. The means of three experiments ± SD are presented (* $p < 0.05$). Statistical significance was checked between the studied probes and controls (no pectin) assumed to be 100%.
